# The Difference Between Polymyxin B and Polymyxin E in Causing Skin Hyerpigmentation

**DOI:** 10.3389/fphar.2021.647564

**Published:** 2021-04-16

**Authors:** Dongna Zou, Haitao Yu, Feifei Li

**Affiliations:** ^1^ Department of Pharmacy, Shandong Provincial Hospital Affiliated to Shandong First Medical University, Jinan, China; ^2^ Department of Pharmacy, Zao Zhuang Municipal Hospital, Zao Zhuang, China; ^3^ Department of Infection, Shandong Provincial Hospital Affiliated to Shandong First Medical University, Jinan, China

**Keywords:** polymyxin B, polymyxin E, skin hyerpigmentation, histamine, melanocytes, oxidative stress, phenylalanine

As we all know, Coronavirus Disease 2019 spread all over the world and had became a public international event of global concern. Among Coronavirus Disease 2019 patients in China, two doctors, Yi Fan and Weifeng Hu, were noticed with their skin pigmentary disorder due to polymyxin B. However, we found that polymyxin E has almost no reports of skin hyperpigmentation, but polymyxin B was reported about skin hyperpigmentation, although the number of relevant reports was small, what causes the difference between the polymyxin B and polymyxin E?

Polymyxin is a general term for a group of basic peptide antibiotics, including A, B, C, D and E mainly. At present, the two commonly used clinically are polymyxin B and polymyxin E.

The mechanism of polymyxin B causing skin hyperpigmentation may include the following aspects:

1. Polymyxin B activates mast cells to release histamine, which binds to the H_2_ receptor on the surface of melanocytes inducing the stimulation of melanin synthesis

Polymyxin B is a mast cell activator that binds to non-selective G protein-coupled receptors on the surface of mast cells, activates subsequent intracellular signaling pathways, and induces mast cells to degranulate and release histamine (Bushby and Green, 1955; Ferry et al., 2002; Morrison et al., 1974; Morrison et al., 1978; Zhan et al., 2019). Histamine is an inflammatory mediator involved in stimulating melanin production. Histamine binds to the H_2_ receptor on the surface of melanocytes in the basal layer to induce the production of cyclic adenosine monophosphate and the activation of protein kinase a in melanocytes, which leads to phosphorylation of members of the cyclic adenylate response element binding protein transcription factor family. Cyclic AMP response element binding protein activates a variety of genes and induces the transcription of a variety of enzymes and proteins related to melanin synthesis. Eventually leads to increased melanin synthesis in the cytoplasm (Yoshida et al., 2020).

Polymyxin E can also release histamine equivalent to polymyxin B (Bushby and Green, 1955), the reason why polymyxin E does not lead to skin hyperpigmentation is not very clear, the difference of amino acid at position 6 between the two drugs and the lower conversion rate of polymyxin E *in vivo* are possible explanation. According to the research, colistin methanesulfonate (CMS) as the prodrug is predominantly cleared by renal excretion, with only a relatively small fraction of the dosage converted to the active antibacterial in renally healthy individuals (Li et al., 2004; Li et al., 2006), it is evident that in patients with moderate to good renal function, administration of a daily dose of colistin base activity (CBA) at the upper limit of the current product-recommended dose range (300 mg CBA per day) was not able to generate plasma colistin concentrations that would be expected to be reliably efficacious (Garonzik et al., 2011). Is the concentration of polymyxin E not enough to activate mast cells to release enough histamine for inducing the stimulation of melanin synthesis? Further study is worthy.

2. The skin inflammation process is related to the activation of melanocytes

Histological and immunohistochemical results of pigmented skin in patients with polymyxin B

treatment showed an abundant melanocyte-pigmented dendritic network. Langerhans cells’ hyperplasia and dermal IL-6 overexpression were also found, presumably for an inflammatory process due to polymyxin B use (Mattos et al., 2016; Li et al., 2020; Wen et al., 2020); At the same time, although hyperpigmented skin did not show signs of inflammationby clinical inspection, under microscopic view the dermis contained mild-to- moderate perivascular inflammatory infiltrate of lymphocytes and histiocytes. Langerhans cells are antigen-presenting cells, which play an important role in skin immunity and inflammation. The proliferation of Langerhans cells in the epidermis of patients with polymyxin B induced-pigmentation indicates that polymyxin B can induce the inflammatory process of the skin. In addition to its known inflammatory effects, IL-6 also inhibits the proliferation and melanogenesis of human melanocytes, When the skin is hyperpigmented, IL-6 is often feedback overexpression which may be for regulation (Jawdat et al., 2004; Mattos et al., 2017). At the same time, studies have shown that mast cell-derived factors (including histamine) can stimulate Langerhans cell migration and are related to the melanin production pathway (Miori et al., 1990).

Matzneller et al. reported that polymyxin E could decrease inflammatory cytokines, including IL-6, in the blood of L lipopolysaccharide-challenged healthy volunteers in a model of human endotoxiemia (Matzneller et al., 2017). And according to the newest report, it was showed that polymyxin E can’t regulate the expression of the inflammatory cytokine IL6, IL6 mRNA expression levels were not changed after administration of polymyxin E (Ubagai et al., 2021).

According to these findings, the effects of polymyxin B and polymyxin E on IL6 are different. We speculate that it may explain the difference in pigmentation between the two drugs.

3. Oxidative stress is also considered to be one of the mechanisms of pigmentation (Zavascki et al., 2016)

Ahmed et al. (2017) studied the effect of polymyxin B on human lung epithelial cells A549, and the results showed that polymyxin B induced oxidative stress and loss of mitochondrial membrane potential. Compared to untreated control cells, after 8 h treatment, the cellular oxidative stress increased around 1.9-fold and 3.8-fold for 1.0 and 2.0 mM of polymyxin B, respectively, which increased up to 2.6-fold and 4.7-fold at 24 h correspondingly. The article also mentioned that the hydrophobicity of the 6-phenylalanine in the structure of polymyxin B and the cationic form under physiological conditions play a key role in cytotoxicity and mitochondrial oxidative stress. This may explain the reason that why the pigmentation had not been found in the patient who used polymyxin E. In the results of polymyxin B nephrotoxicity studies, it was found that mitochondrial stress response and the production of reactive oxygen species were found in renal tubular cells treated with polymyxin B (Azad et al., 2015). Reactive oxygen species such as NO can induce the activation of guanylate cyclase and enhance the expression of tyrosinase gene to increase melanin production (Sasaki et al., 2000).

4. Phenylalanine increases melanin synthesis (Martindale, 2014)

Compared with polymyxin E, polymyxin B has a different structure at position 6. Polymyxin B is phenylalanine at position 6, and polymyxin E is leucine. According to Martindale records, vitiligo can be treated with phenylalanine, and more than 60% of patients have skin pigmentation during the treatment process (Kopple et al., 2007). This could be explained by that phenylalanine is hydroxylated in the body to form tyrosine (Chang et al., 2009), which is a non-essential amino acid in the human body and the main raw material for the synthesis of melanin (Rzepka et al., 2016). And we have not seen any reports of leucine-induced skin hyperpigmentation, that maybe another reason why polymyxin E-related pigmentation has not been reported.

The nephrotoxicity and neurotoxicity induced by polymyxin B have been proved to be dose-dependent (John et al., 2017), but there is no direct evidence that polymyxin B-induced skin hyerpigmentation is a dose-dependent adverse reaction. But it was speculated that acute kidney injury (AKI) with lower creatinine clearance may be an important factor for polymyxin B-induced pigmentary disorder (Zheng et al., 2018; Lu and Hou, 2020). In addition, neonates and infants were more likely to suffered from skin hyerpigmentation after polymyxin B administration than adults, it was reported that 16 infants in ICU generalized skin hyperpigmentation in premature infants receiving polymyxin B (Shih and Gaik, 2014), and it was also noted generalized skin hyperpigmentation among neonates receiving IV polymyxin B (Gothwal et al., 2016), according to these findings, we speculate that it may be related to their immature kidney function leading to the cumulation of polymyxin B (Gothwal et al., 2016; Li et al., 2020), as polymyxin B is excreted through the kidney, while the incidence of skin hyperpigmentation were 15% or 8% of adult patients (Mattos et al., 2016; Mattos et al., 2017).

It should be pointed out that there have been no reports of inhalation of polymyxin B-induced skin hyperpigmentation so far, the administration for all patients suffered from skin hyerpigmentation induced by polymyxin B were intravenous (Knueppel and Rahimian, 2007; Shih and Gaik, 2014; Zavascki et al., 2015; Gothwal et al., 2016; Mattos et al., 2016; Zavascki et al., 2016; Lahiry et al., 2017; Mattos et al., 2017; Zheng et al., 2018), which is worthy of further discussion on the relationship between the administration and adverse drug reaction.

According to the findings above, we speculate boldly that polymyxin B needs to reach a certain concentration in blood to cause pigmentation and that reducing the dosage may be an effective way to prevent skin hyperpigmentation, however, reducing the dosage of polymyxin B may be likely to reduce the efficacy and even lead to bacterial resistance. It should be point out that there is no definite evidence that the occurrence of pigmentation is related to the increased concentration of polymyxin B in blood, and there is no report of dosage adjustment after the occurrence of skin pigmentation.

Regarding the difference between polymyxin B and polymyxin E in causing skin pigmentation, further research is needed in the future, and further research is needed on how to prevent and treat polymyxin B-induced skin pigmentation. However, the current related reports can remind us that we should pay attention to monitoring related adverse reactions when applying polymyxin B. If skin hyerpigmentation occurs, provide corresponding psychological counseling to the patient, or take corresponding treatment measures such as laser cosmetic therapy and topical whitening agents, or adjust the dosing regimen if necessary.
